# Induction of apoptosis in MCF-7 cells by the hemagglutinin-neuraminidase glycoprotein of Newcastle disease virus Malaysian strain AF2240

**DOI:** 10.3892/or.2013.2573

**Published:** 2013-06-27

**Authors:** MOHAMED GHRICI, MOHAMED EL ZOWALATY, ABDUL RAHMAN OMAR, AINI IDERIS

**Affiliations:** 1 Department of Veterinary Pathology, Veterinary Clinical Studies, Faculty of Veterinary Medicine, University Putra Malaysia, Malaysia; 2 Department of Microbiology, Veterinary Clinical Studies, Faculty of Veterinary Medicine, University Putra Malaysia, Malaysia; 3 Laboratory of Vaccines and Immunotherapeutics, Institute of Bioscience, University Putra Malaysia, 43400 UPM Serdang, Selangor Darul Ehsan, Malaysia

**Keywords:** Newcastle disease virus AF2240, hemagglutinin-neuraminidase glycoprotein, MCF-7, apoptosis

## Abstract

Newcastle disease virus (NDV) exerts its naturally occurring oncolysis possibly through the induction of apoptosis. We hypothesized that the binding of the virus to the cell via the hemagglutinin-neuraminidase (HN) glycoprotein may be sufficient to not only induce apoptosis but to induce a higher apoptosis level than the parental NDV AF2240 virus. NDV AF2240 induction of apoptosis in MCF-7 human breast cancer cells was analyzed and quantified. In addition, the complete HN gene of NDV strain AF2240 was amplified, sequenced and cloned into the pDisplay eukaryotic expression vector. HN gene expression was first detected at the cell surface membrane of the transfected MCF-7 cells. HN induction of apoptosis in transfected MCF-7 cells was analyzed and quantified. The expression of the HN gene alone was able to induce apoptosis in MCF-7 cells but it was a less potent apoptosis inducer compared to the parental NDV AF2240 strain. In conclusion, the NDV AF2240 strain is a more suitable antitumor candidate agent than its recombinant HN gene unless the latter is further improved by additional modifications.

## Introduction

Newcastle disease virus (NDV), known as avian paramyxovirus of serotype 1 (APMV-1), is assigned as the type species member of the genus *Avulavirus* belonging to the *Paramyxoviridae* family within the order *Mononegavirales*(1). NDV is an enveloped virus which consists of a nucleocapsid harboring a non-segmented, negative sense single-stranded RNA genome consisting of 6 transcriptional units in the order of 3′-NP-P-M-F-HN-L-5′ which encodes at least 6 major proteins, i.e. the nucleocapsid (NP), the phosphoprotein (P), the matrix protein (M), the large polymerase protein (L), and 2 types of trans-membrane glycoproteins, the hemagglutinin-neuraminidase (HN) and the fusion (F) proteins (2,3). Two additional proteins, V and W, are expressed by mRNAs, which are derived from the P gene via RNA editing (4).

The natural reservoir of NDV is wild birds and NDV is an economically considerable avian pathogen causing extensive morbidity, mortality and losses in the poultry industry in several countries (5). On the other hand, NDV is non-pathogenic to humans and its oncotherapeutic benefits have been used in preclinical studies making it a promising non-conventional oncovirotherapeutic agent (6). NDV is as highly suitable as an oncolytic agent and is also considered a potential agent in the treatment of cancer as it selectively kills tumor cells (7). AF2240 is a viscerotropic-velogenic NDV (VVNDV) Malaysian strain isolated in the 1960s during a local field outbreak (8).

The HN glycoprotein protrudes from the viral envelope in the non-fusion virion and in NDV-infected cells where it is expressed at the cell surface (9). HN glycoprotein is a multifunctional protein; it plays a major role in NDV infection, pathogenesis and is responsible for the immunogenic properties of NDV (10). HN protein is responsible for NDV attachment to sialic acid receptor and it contains the neuraminidase (sialidase) activity (11).

NDV is known as a naturally occurring oncolytic virus and it is cytotoxic against several types of human tumor cells (12,13). NDV replicates selectively in tumor cells but not in the normal cells (14). The oncolytic activity of NDV has been exploited in the development of various types of antitumor vaccines (15) and is mediated by apoptosis induction (14,16,17). The oncolytic- and apoptotic-induced activities of NDV have been well characterized but their molecular mechanisms are not fully understood. Earlier investigators suggested that NDV induced apoptosis through signal molecules such as IFN-α and TNF-α (18) and TRAIL (17). TNF-α and TRAIL are well known apoptosis inducers and simultaneously exert an antitumor activity (19,20).

Little is known about the HN glycoprotein oncolytic activity. As for parental NDV, HN oncolytic activity may be mediated by apoptosis. HN glycoprotein was shown to induce apoptosis in human hepatoma SMMC-7721 cells (21). Similar to their parental NDV, the molecular mechanism of HN apoptosis or oncolysis induction has yet to be elucidated. The antitumor properties of HN may be related to IFN-α and TRAIL which were induced by HN expression in human blood mononuclear cells (22). Based on its binding and neuraminidase activities, the HN protein was able to activate adhesion molecules and increase the tumor cytotoxic T lymphocytes (CTL) responses (23). The antitumor activity of HN appears to be dependent on its cell surface localization within the tumor cell. A membrane anchored HN showed enhanced antitumor effect compared to the cytoplasmic or the secreted HN protein (24). *In vivo* expression of HN protein reduced tumor growth and stimulated innate antitumor activity in a mouse model (25). The expression of HN protein of an Indian NDV strain was shown to induce apoptosis in chicken embryo fibroblast (CEF) cells (26).

The current study is part of a major project aimed at developing an anticancer vaccine based on NDV AF2240 strain for the treatment of human breast cancer. However, it is imperative to understand the oncolytic mechanism of NDV AF2240 strain which is a prerequisite for efficient development of a cancer vaccine candidate. Little is known about NDV AF2240 strain oncolytic activity. Only *in vitro* cytotoxicity studies of NDV AF2240 have been carried out and it was found that NDV AF2240 strain induced apoptosis in a number of tumor cell lines including WEHI-3B leukemic (27), brain tumor (28), HT-29 human colon adenocarcinoma, HCT-11 Bax and wt colorectal carcinoma cells (29). The oncolytic activity of NDV AF2240 strain and several other local NDV strains (C, Ijuk, S, F and V4) were screened on tumor cell lines including CEM-SS (T-lymphoblastic leukemic cells) and HT-29 based on an MTT cytotoxic assay and it was found that NDV AF2240 strain was more cytotoxic to tumor cells than other strains (30).

The molecular mechanism of NDV AF2240-induced apoptosis is not fully understood with the exception that NDV AF2240 strain induced conformational changes of Bax protein which in turn is translocated from the cytoplasm to mitochondria and this leads to the release of cytochrome *c* in the cytoplasm (31). However, neither the signaling mechanism leading to the conformational changes of Bax nor the type of apoptotic stimuli responsible for this conformational change were identified. The current study is the first to demonstrate that the expression of NDV AF2240 strain’s HN alone induced apoptosis in MCF-7 cells. Based on similar reported studies, we hypothesized that the expression of HN glycoprotein of NDV AF2240 strain may not only induce apoptosis but it may also be a stronger inducer of apoptosis in MCF-7 cells than the parental virus.

The objective of the present study was to demonstrate whether HN expression alone induced apoptosis in MCF-7 cells and to compare the potency of both HN glycoprotein and the parental NDV AF2240 strain in inducing apoptosis in MCF-7 cells in order to select the most suitable antitumor candidate for future investigations.

## Materials and methods

### Experimental design

The complete HN gene of NDV AF2240 strain was amplified, cloned and expressed at the MCF-7 cell surface. The induction of apoptosis by both recombinant HN and parental NDV AF2240 were demonstrated by flow cytometry analysis and were statistically analyzed. The potency of apoptosis induction by the recombinant HN and NDV AF2240 strain was analyzed.

### Cell and virus

Human breast carcinoma MCF-7 cells (ATCC^®^ no. HTB-22™) were cultured in RPMI-1640 tissue culture medium supplemented with 10% fetal bovine serum (FBS) and 1% of antibiotic-antimycotic. The cells were maintained at 37ºC in 5% CO_2_ atmosphere. The medium, serum and antibiotics were purchased from Invitrogen Life Technologies (Carlsbad, CA, USA).

Virus stock was prepared by propagation in 9-day old embryonated SPF eggs, followed by purification as previously described (32). The virus was titrated by hemagglutination assay and stored as single-use aliquots at −80ºC for all experiments.

### NDV AF2240 strain-induced apoptosis

#### Flow cytometry analysis

MCF-7 cells (5×10^6^) cultured in 25 cm^2^ tissue culture flasks were infected with various concentrations of NDV AF2240 strain of including 50, 100, 250 and 500 hemagglutination units (HAUs). After 1 h adsorption, the virus inoculums were removed and fresh RPMI-1640 medium was added. The infected cells were incubated for 48 h at 37ºC in the presence of 5% CO_2_ atmosphere. NDV AF2240 strain-induced apoptosis was assessed using flow cytometry according to a previously described method (33). At the end of the incubation time, both adherent cells and supernatant were processed by 2 successive centrifugations of 1,000 rpm for 10 min, followed by fixation in 80% cold ethanol for 2 h at 4ºC. After 3 successive centrifugations at 1,000 rpm for 10 min, the cells were incubated for 5 min at 4ºC in 1X phosphate-buffered saline (PBS) buffer containing 10 mM Triton X-100 and 50 μg/ml of RNase A (Invitrogen Life Technologies). Following centrifugation, the cells were incubated for 30 min at 4ºC in the dark in 1 ml of 1X PBS buffer containing 5 μg/ml propidium iodide (PI) (BioResource International, Morrisville, NC, USA). Apoptosis was assessed by flow cytometry analysis. The stained cells were then analyzed with a CyAn ADP (Beckman Coulter, Brea, CA, USA) flow cytometer. The data were analyzed with Summit v4.3 software (Beckman Coulter).

#### Mitochondrial transition pore assay

MCF-7 cells (10^5^) grown in chamber slides (Nalge Nunc International, Rochester, NY, USA) were infected with 250 HAUs of NDV AF2240 strain. After 1 h virus adsorption, the cells were incubated for 1 h at 37ºC in the presence of 5% CO_2_ atmosphere. The non-infected and infected MCF-7 cells were processed for the detection of the activation of mitochondrial transition pore opening using Image-iT live mitochondrial transition pore assay kit according to the manufacturer’s protocol (Molecular Probes; Invitrogen Life Technologies, Carlsbad, CA, USA). The cells were viewed under a fluorescence microscope (Leica DMRA II, Germany).

#### Reverse transcription-polymerase chain reaction (RT-PCR) amplification of HN gene

Total RNA was extracted from NDV AF2240 strain-infected allantoic fluid of specific-pathogen-free embryonating chicken eggs using TRI Reagent according to the manufacturer’s instructions (Promega Corporation, Madison, WI, USA). The primer set for the amplification of the complete HN gene was designed using the Primer premier 5.0™ software and based on the published sequence of NDV AF2240 strain (accession number X79092). Two restriction enzyme sites *Sal*I and *Sac*II were included in the primer set. The primer set sequences were: HNSF, 5′-AAT CCG CGG ATC ATG GAC CGT GCA GTT AG-3′ and HNSR, 5′-GGG GTC GAC CTC TCA TGG TTG ACT CAA-3′. The amplification of the complete HN gene was performed using access RT-PCR System (Promega Corporation, Madison, WI, USA). The HN gene was amplified in a reaction mixture containing 1.5 mM MgS0_4_, 10 mM each of dNTP mix, 0.2 units RNase inhibitor, 5 units of *Avian myeloblastosis virus* (AMV) reverse transcriptase, 5 units of *Tf1* DNA polymerase, and 0.5 μM of each primer. A total RNA of 420 ng was added. The reaction mixture was first incubated for 45 min at 42ºC followed by 95ºC for 5 min. Then, 40 cycles of 94ºC for 45 sec, 65ºC for 1 min and 70ºC for 1 min were carried out. A last step of 72ºC for 5 min was added. The amplified HN fragment was fractionated on 1% Tris-borate-EDTA agarose gel, stained with ethidium bromide solution (50 ng/ml) and analyzed on gel alpha imaging system (Alpha Innotech Corp., San Leandro, CA, USA).

#### Plasmid constructs

The amplified HN fragments were purified using the wizard SV gel and PCR clean up system according to the manufacturer’s instructions (Promega Corporation). The purified HN fragment was cloned into pCR 2.1 vector using the TOPO TA Cloning^®^ kit according to the manufacturer’s instructions (Invitrogen Life Technologies). The positive recombinants were analyzed by *Eco*RI restriction enzyme digestion. The recombinant pCR 2.1-HN and the expression vector pDisplay were purified by using the pure yield plasmid Midiprep System (Promega Corporation) and double digested with *Sal*I and *Sac*II. The linearized HN fragment was cloned into double digested pDisplay vector and incubated for 1 h at 16ºC. The orientations of the positive recombinants were examined by double digestion with *Sal*I and *Sac*II and the authenticity of the positive recombinant was confirmed by DNA sequencing.

#### Transfection

MCF-7 cell population of 0.5×10^5^ was plated in 500 μl of RPMI-1640 free antibiotic-antimycotic tissue culture medium. MCF-7 cells were cultured to ~80% confluency at the time of transfection. For each transfection, 0.3–1.2 μg of the recombinant pDisplay-HN diluted in 100 μl of Opti-MEM^®^ I reduced serum medium was transfected into MCF-7 cells using 1.25–10 μl of Lipofectamine^®^ LTX Reagent according to the manufacturer’s instructions (both from Invitrogen Life Technologies). The transfected cells were incubated for 48 h at 37ºC in a 5% CO_2_ incubator before assay of HN expression and apoptosis induction.

#### Immunofluorescence assay

The detection and localization of HN glycoprotein was carried out using indirect immunofluorescence assay. Briefly, MCF-7 cells were transfected as previously described and incubated for 48 h at 37ºC in a 5% CO_2_ atmosphere. The glass slide containing the transfected MCF-7 cells was removed from the Lab-Teck chamber slide and rinsed with 1X PBS buffer. The cells were fixed in cold acetone for 10 min at room temperature. A dilution of 1:200 of chicken polyclonal anti-NDV AF2240 serum prepared previously was added to the fixed cells, incubated at room temperature for 30 min and rinsed a few times with 1X PBS buffer. A fluorescein-labeled affinity purified antibody to chicken IgG (Kirkegaard & Perry Laboratories, Gaithersburg, MD, USA) diluted at 1:100 was added to the cells and incubated at room temperature for 30 min. The cells were rinsed with 1X PBS buffer and dried. Finally, 1 drop of Antifade Solution (Chemicon International, Temecula, CA, USA) was added and cells were visualized using a fluorescence microscope (Leica DMRA II).

#### Flow cytometry analysis

The detection of HN induction of apoptosis was carried out as previously described (33). Briefly, MCF-7 cells were transfected as described above with 0.3, 0.6 and 1.2 μg of recombinant pDisplay-HN and transfected cells were incubated for 48 h at 37ºC in a 5% CO_2_ atmosphere. Both the transfected cells and the supernatant were harvested and processed for flow cytometry analysis as described above.

#### Statistical analysis

Data were analyzed and expressed as means ± SD. Statistical analysis was performed using one-way analysis of the percentage of apoptosis by the recombinant pDisplay-HN. The comparison for the pairs was carried out by the Tukey-Kramer HSD test from flow cytometry assays.

## Results

### NDV AF2240-induced apoptosis in MCF-7 cells

**Figure 1:**
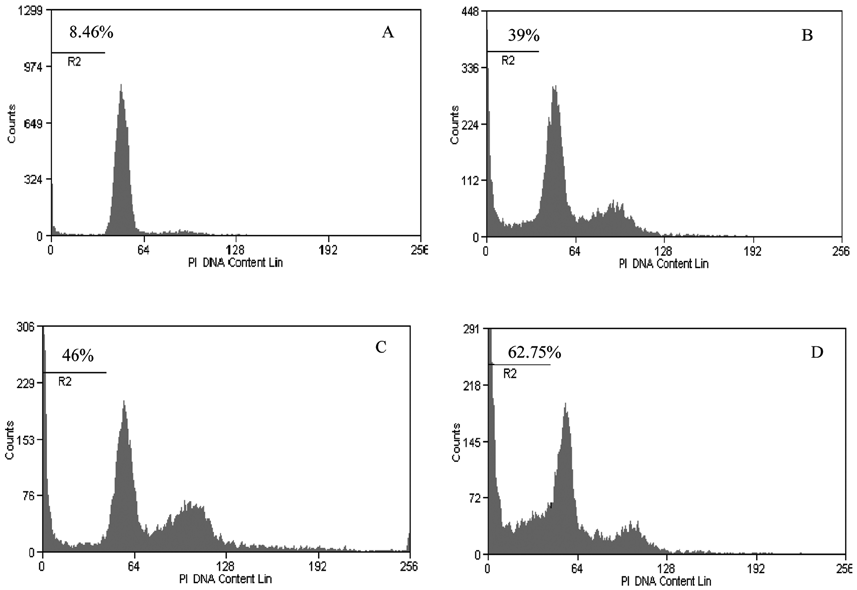
Newcastle disease virus (NDV) AF2240 induced-apoptosis in MCF-7 cells is dose-dependent. (A) Image shows small physiologic amounts of apoptosis found in non-infected MCF-7 cells. MCF-7 cells were infected with various titers of the viral strain and cells were incubated for 48 h in a 5% CO_2_ atmosphere. At the end of the incubation time, the rate of apoptosis induced by NDV strain AF2240 was assessed by flow cytometry analysis which showed 39% of apoptosis in cells infected with 50 hemagglutination units (HAUs) of NDV AF2240 (B) and 46% of apoptosis when cells were infected with 100 HAUs (C). (D) Infection with 250 HAUs of NDV strain AF2240 induced 62.75% of apoptosis.

**Figure 2:**
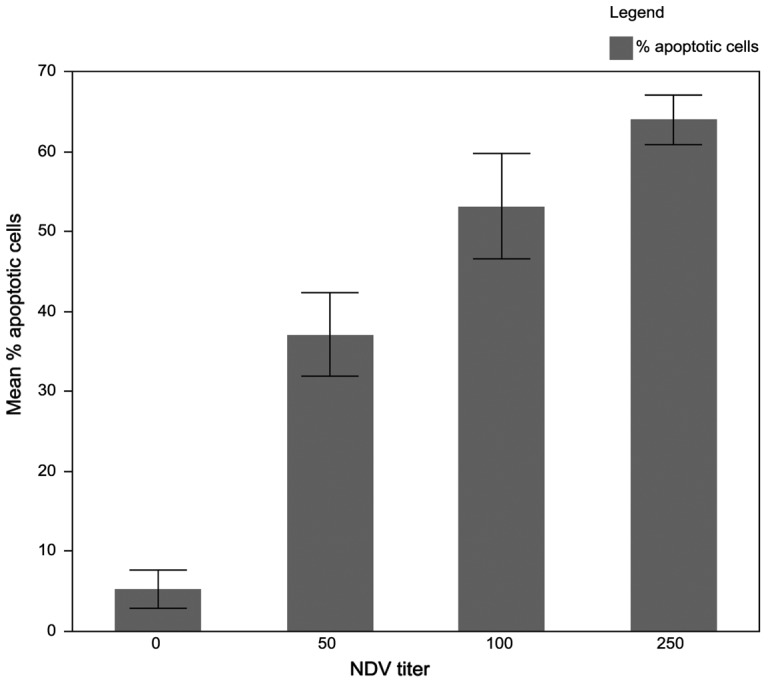
Effect of increase in Newcastle disease virus (NDV) strain AF2240 titers on the percentage of apoptotic MCF-7 cells. MCF-7 cells were infected with various concentrations of NDV and incubated for 48 h. The results were expressed as the mean percentages of apoptotic cells assessed directly by flow cytometry. Each value represents the average ± the standard deviation of triplicate cultures. Error bars indicate standard deviations of the means. Each error bar is constructed using 1 standard deviation from the mean.

**Figure 3:**
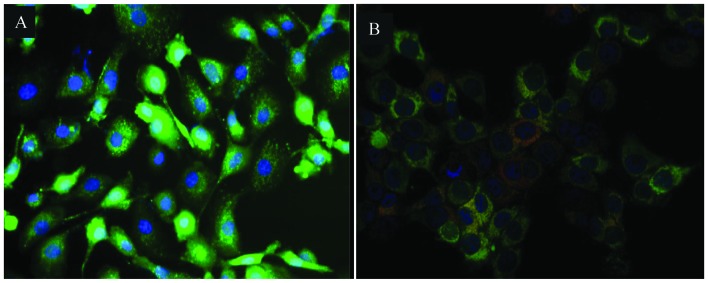
Detection of mitochondrial permeability transition pore opening. MCF-7 cells were infected with 250 hemagglutination units (HAUs) of Newcastle disease virus (NDV) strain AF2240. After 1 h of incubation at 37ºC in a 5% CO_2_ atmosphere, the mock and infected cells were rinsed twice with a modified HBSS buffer, then labelled with a solution containing mitotracker red CMXROS and incubated for 15 min at 37ºC in a 5% CO_2_ atmosphere. Finally, the cells were rinsed and visualized under a fluorescence microscope. (B) MCF-7 infected cells displayed red and yellow fluorescence which is a typical characteristic of the mitochondrial permeability transition pore opening activation. (A) This latter was not activated in non-infected MCF-7 cells which maintained the green fluorescence in the cytoplasm in the form of the comet. (A) The nucleus was stained blue with Hoechst.

The result of apoptosis induction using various titers of NDV AF2240 strain revealed that 250 HAUs induced the highest apoptosis level in MCF-7 cells (Fig. 1). At higher titer of 500 HAUs and above, the viral AF2240 strain was more cytotoxic to MCF-7 cells grown in 25 cm^2^ tissue culture flasks in which the cells detached from the surface of the flask in less than 24 h post infection (pi). The apoptosis induction increased from 37% obtained after infection with 50 HAUs to ~63% of dead cells with infection with 250 HAUs. The increase of the percentage of apoptotic cells was significant for all titers of NDV as compared to negative control (p<0.0001), as shown in Fig. 2. The induced apoptosis was dose-dependent. There was a positive correlation between the dose of NDV and the percentage of apoptotic cells, suggesting that NDV AF2240 strain kills 100% of MCF-7 cells if the dose is increased. The NDV AF2240 strain dose of 250 HAUs was selected based on its strong induction of apoptosis for the detection of mitochondrial permeability transition pore activation. MCF-7 cells infected with 250 HAUs of NDV AF2240 showed a red fluorescence in the form of a comet which is a typical characteristic of mitochondria permeability transition pore opening activation while only green fluorescence was observed in non-infected MCF-7 cells (Fig. 3).

### Amplification of the whole HN gene

**Figure 4:**
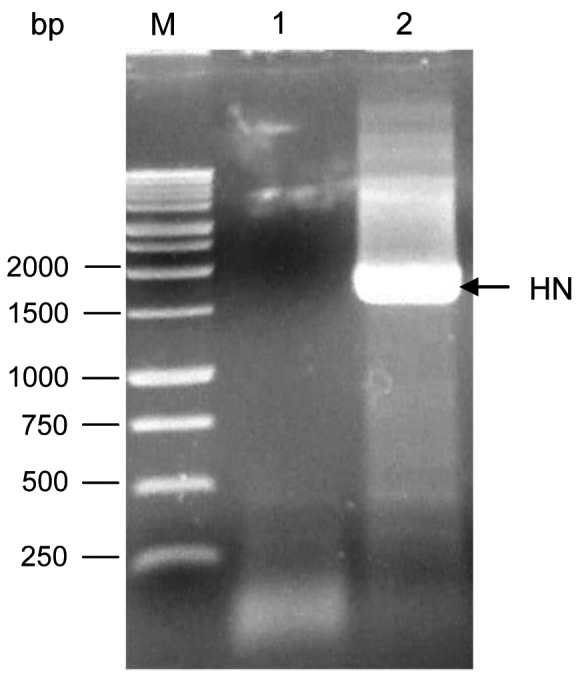
Agarose gel electrophoresis of RT-PCR amplification of the complete hemagglutinin-neuraminidase (HN) gene of Newcastle disease virus (NDV) strain AF2240. The amplified HN gene with the size of ~1.8 kb was fractionated on agarose gel and stained with ethidium bromide (lane 2) while amplification from total RNA extracted from non-infected MCF-7 cells (lane 1) was negative. Lane M represents 1 kb DNA ladder (Promega Corporation, Madison, WI, USA).

The whole HN gene was amplified using RT-PCR at the expected size of ~1.8 kb as shown in Fig. 4. To avoid introducing deleterious mutations, the HN DNA fragment was fractionated on agarose gel electrophoresis without exposing it to ethidium bromide and UV light. Then, the HN DNA fragment was purified and stored at −20ºC.

### Plasmid constructs

**Figure 5:**
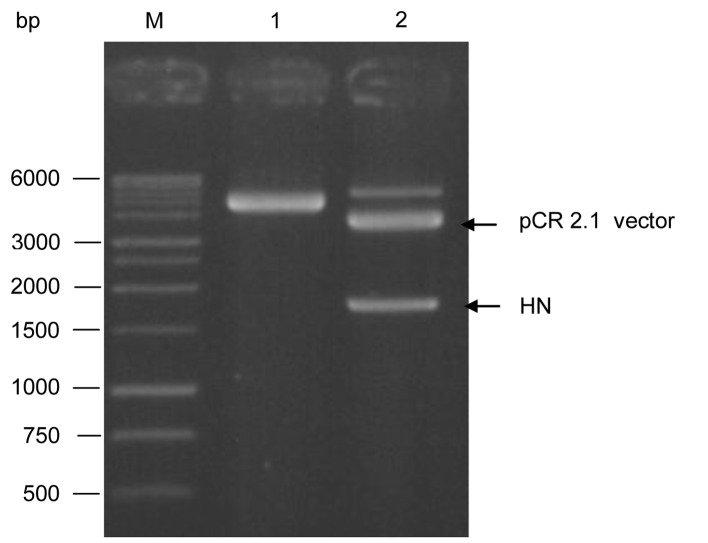
Agarose gel electrophoresis of the positive recombinant pCR 2.1/HN and pDisplay expression vector. Both the positive recombinant pCR 2.1/HN and pDisplay vector were double digested with the restriction enzymes *Sal*I and *Sac*II which released the hemagglutinin-neuraminidase (HN) fragment from the pCR 2.1 vector (lane 2). The double digested pDisplay vector was fractionated at the size of 5.3 kb (lane 1). Lane M represents 1 kb DNA ladder (Promega Corporation, Madison, WI, USA).

**Figure 6:**
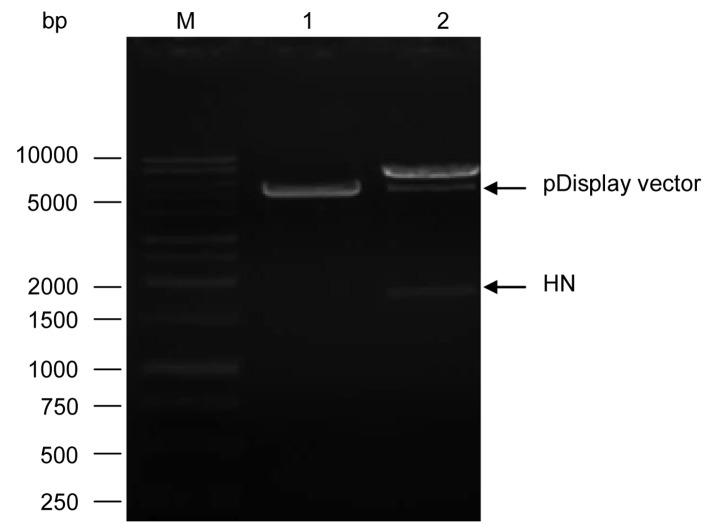
Agarose gel electrophoresis of the cloning of hemagglutinin-neuraminidase (HN) gene into pDisplay expression vector. The positive recombinant pDisplay/HN was analyzed by double digestion using restriction enzymes *Sal*I and *Sac*II which released the HN gene of size 1.8 kb (lane 2). pDisplay vector was also double digested with both enzymes (lane 1). Lane M represents 1 kb DNA ladder (Promega Corporation, Madison, WI, USA).

The pure HN DNA fragment (1 μl) was first cloned in pCR 2.1-TOPO cloning vector. The positive recombinant pCR 2.1-HN was analyzed by *Eco*RI restriction enzyme as shown in Fig. 5. The pCR 2.1-HN and the pDisplay expression vector were purified and double digested with *Sal*I and *Sac*II restriction enzymes. A ratio of vector: insert of 1:5 was the most suitable for a successful cloning of the HN fragment into pDisplay expression vector and the successful recombinant pDisplay-HN was confirmed by the release of HN gene by double digestion with the restriction enzymes *Sal*I and *Sac*II which released the HN fragment as shown in Fig. 6. The sequencing of the cloned HN gene revealed a correct in-frame cloning.

### Expression of HN gene induces apoptosis in MCF-7 cells

**Figure 7:**
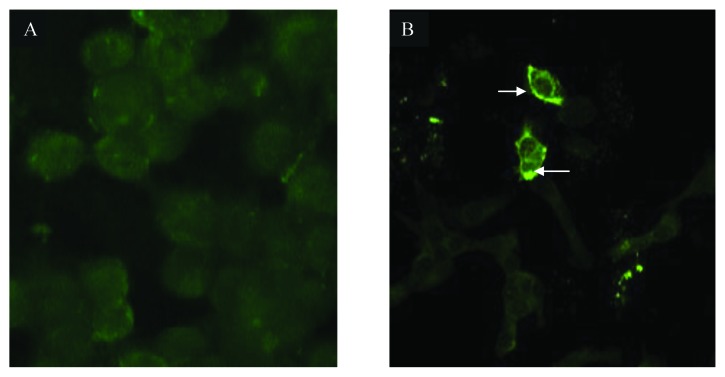
Expression of the recombinant hemagglutinin-neuraminidase (HN) at MCF-7 cell’s surface membrane. MCF-7 cells were transfected with 1.2 μg of recombinant pDisplay/HN. (A) No immunofluorescence was detected in mock-infected MCF-7 cells. (B) HN expression and its specific localization (arrows) at the cell surface membrane of the transfected MCF-7 cells were analyzed using indirect immunofluorescence at 48 h post-transfection.

**Figure 8:**
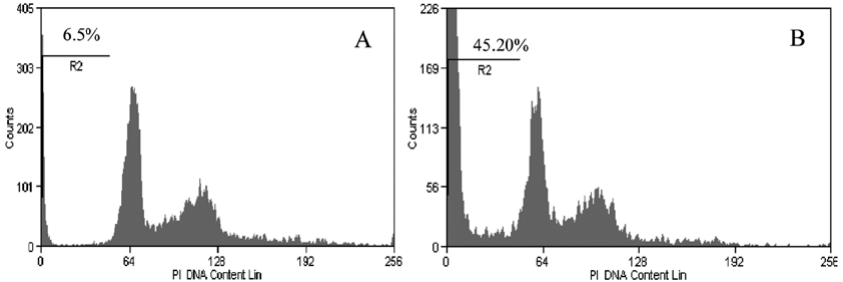
Recombinant hemagglutinin-neuraminidase (HN) expression induced apoptosis in transfected MCF-7 cells. MCF-7 cells transfected with 1.2 μg of the recombinant pDisplay/HN were analyzed using flow cytometry after 48 h of incubation. HN expression in MCF-7 cells induced 45% of apoptosis (B) compared to 6.5% of normal physiological death in non-transfected MCF-7 cells (A). Marker R2 in the histogram represents the percentage of apoptotic cells. Experiments were repeated in triplicate.

**Figure 9:**
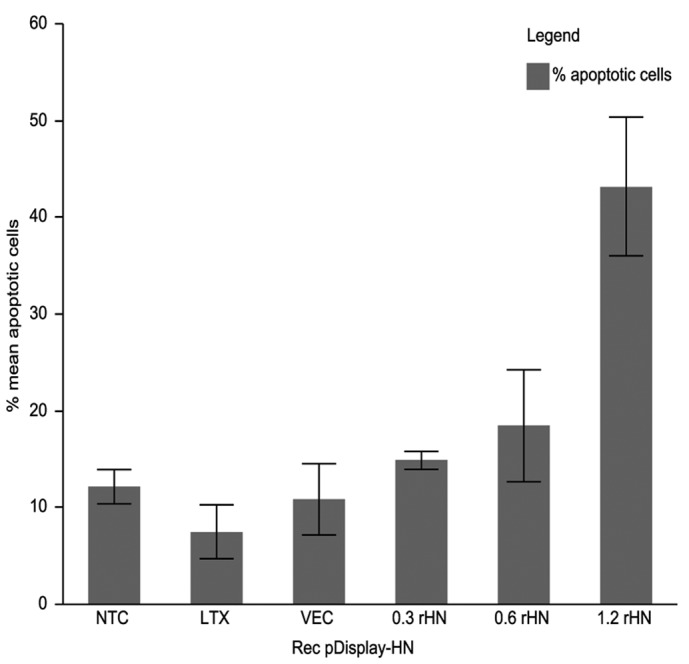
Induction of apoptosis in MCF-7 cells by recombinant hemagglutinin-neuraminidase (HN) gene. MCF-7 cells were transfected with various amounts of recombinant of pDisplay/HN and cells were analyzed using flow cytometry at 48 h post transfection. Apoptosis induction was found to be dose-dependent. MCF-7 cells were also transfected with LTX reagent only and with pDisplay vector only. NTC, non-transfected MCF-7 cells. LTX, Lipofectamine^®^ LTX transfectant reagent. VEC, transfection with vector only. rHN, recombinant pDisplay/HN. Each bar represents the results of 3 independent experiments. Error bars indicate the standard of deviation.

It was revealed that apoptosis induced by NDV AF2240 strain in MCF-7 cells may be mediated by HN protein expression alone. This hypothesis was investigated in 2 steps. First, the positive recombinant pDisplay-HN harboring the complete HN gene was transfected into MCF-7 cells. The expression and localization of HN protein were detected by indirect immunofluorescence assay as shown in Fig. 7. HN expression-induced apoptosis was confirmed and quantified using flow cytometry. The recombinant pDisplay-HN at a concentration of 1.2 μg induced the highest level of apoptosis as shown in Fig. 8. The percentage of apoptotic cells increased from ~15% with transfection of 0.3 μg of recombinant pDisplay-HN to 43% with transfection of 1.2 μg of recombinant pDisplay. The significant increase of apoptosis was induced with 1.2 μg of recombinant HN (p<0.0001). Lower concentrations of 0.3 and 0.6 μg recombinant HN did not induce any significant increase of apoptosis. Lipofectamine LTX treatment of MCF-7 cells and transfection of MCF-7 cells with pDisplay vector only had no significant effect on apoptosis induction as shown in Fig. 9. The apoptosis induced by recombinant HN was dose-dependent.

## Discussion

NDV strain AF2240 is known to induce apoptosis in a number of human tumor cell lines, but induction of apoptosis in MCF-7 cells was not well documented. In the present study, we first evaluated the extent of NDV AF2240 strain-induced apoptosis using flow cytometry analysis. Second, we confirmed NDV AF2240 induction of apoptosis via mitochondrial permeability transition pore opening assay. The mechanisms of apoptosis induction and regulation are not fully elucidated. This is due, in part, to the existence of a wide variety of stimulatory factors (34).

The majority of apoptosis research is focused on these stimulatory and initiating signals due to their significance in apoptosis modulation. Apoptosis is a physiological process used as a defence or a self-destructive mechanism against infection and neoplasm in which infected or tumor cells are eliminated by a cell death suicide (35,36). Imbalance in apoptosis causes diseases either when excess apoptosis occurs, as in the case of degenerative diseases, or when a failure or decrease in apoptosis happens, as in the case of autoimmune diseases and cancer (37). Most of the antitumor research takes advantage of the apoptosis mechanism (38).

The antitumor activity of several NDV strains is well documented (7,15). Some of these strains were tested in a clinical setting while others are in an advanced clinical trial (7,15,39–44).

In this study, we focused on NDV AF2240 strain as a continuation of its previously reported cytotoxic activities *in vitro* and apoptotic activity in tumor cell lines (27,28). However, little is known about the molecular mechanism of NDV AF2240-induced apoptosis. The role of NDV AF2240 gene(s) in apoptosis has not been investigated. Earlier studies showed that HN glycoprotein mediates the expression of TNF-α and TRAIL (17,22). It appears that TRAIL-induced apoptosis is a general phenomenon since it was shown in numerous viruses including, but not exclusive to, avian influenza (45), reovirus (46), measles (47) and respiratory syncytial virus (48). However, the molecular mechanism of HN induction of these cytokines remains to be fully elucidated. HN may not be the only NDV gene inducing death ligands; these latter can be induced by cellular stress proteins which can also be induced by NDV infection (49). In addition, HN glycoprotein may share similar effects with other viral envelope proteins which were shown to induce cytotoxicity via apoptosis induction such as HIV gp120 (50) and sigma-1 attachment protein of type 3 reovirus (51). HN glycoprotein of NDV is a well-known mediator of cytotoxicity (10).

In another study, HN expression was shown to induce apoptosis in normal chicken embryo fibroblast (CEF) cells (26). We were able to induce a higher level of apoptosis (45.20%) with only a small amount of recombinant HN at 48 h post infection (pi) compared to the low level of apoptosis induction (4.9%) induced using 5 μg of recombinant HN at 96 h pi as previously reported (26). This difference in apoptosis level may be due to differences in the cell type, the use of different expression systems and different origin of the HN glycoprotein derived from different NDV pathotypes. The expression system may have played a greater role in inducing a high level of apoptosis as it was demonstrated in a previous study in which the Semliki forest virus (SFV) based expression system harboring NDV HN gene which generated a stronger expression of HN protein which in turn induced 90% of cell death (52).

In the present study, the lower level of apoptosis induced by recombinant HN may be attributed to several factors including, in particular, the pDisplay expression system used. The induced apoptosis by HN protein was found to be dose-dependent to a certain level but not as potent as the parental virus NDV AF2240 strain-induced apoptosis. This low level of apoptosis induced by the recombinant HN may be partially explained by the insufficient amount of recombinant HN DNA used to transfect MCF-7 cells. This is due to the limiting factor of the transfection reagent used. It was not possible to increase the level of apoptosis indefinitely by increasing the amount of the recombinant HN DNA. An increased amount of recombinant HN DNA requires an increased level of transfection agent which becomes too toxic to MCF-7 cells. Second, recombinant HN DNA cannot transfect every MCF-7 cell since it is not an infectious or self replicating agent. These 2 shortcomings did not apply for NDV AF2240 strain-induced apoptosis. NDV AF2240 is not only highly infectious but it is also possible to increase the amount of the virus indefinitely to induce a massive and complete cell death of the infected MCF-7 cells. In a clinical setting, this fact has potential benefits in cancer treatment as long as other limiting factors such as the development of antitumor resistance, virus escape mutant and anti-NDV antibodies are kept in control. The administration of massive amount of NDV has resulted in encouraging outcomes in the past (39).

The use of parental NDV AF2240 strain in inducing apoptosis was more efficient compared to recombinant HN protein. This finding is in agreement with previous findings that NDV-induced apoptosis depends on the virus particle itself (17,22,53), indicates that apoptosis is NDV dose-dependent as found in the present study. Our observation that apoptosis level is NDV dose-dependent is in agreement with previous findings (54). However, replication within the tumor cell may have an additive greater effect in inducing a higher level of apoptosis. In agreement with this suggestion, it was reported that one infectious NDV particle was able to kill at least 10^4^ tumor cells in 2–3 days (15). The reason that NDV particle induced a higher level of apoptosis than recombinant HN protein may be due to the contribution of other NDV genes. In fact, NDV M protein was shown to have an additive role in apoptosis induction (55). Hence, the involvement of other NDV genes either alone or in synergy with cellular genes cannot completely be excluded.

It is known that NDV infection stimulates several cellular genes and the production of chemokines and cytokines (53). The involvement of cytokines such as TRAIL are well known apoptosis inducers (20). In addition, this difference in apoptosis induction level can be explained by the difference in the binding avidity between parental NDV AF2240 and recombinant HN protein as in the case of IFN-α induction where it was suggested that IFN-α induction by recombinant HN was 70% lower than the induction by the complete NDV virion (22). The binding avidity difference may be due to the overall structural organization of the HN protein within the virion and when HN is expressed alone. It is not known whether all HN glycoproteins irrespective of their origin of NDV strains and pathotypes induced apoptosis or induced similar levels of apoptosis in a particular cell type or in all cell types. In other viruses, such as rabies virus, only G protein of non-pathogenic rabies virus (RV) strain ERA induced apoptosis while G protein from highly neurotropic RV strain CVS did not induce apoptosis (56).

Whether the complete HN gene or part of it is required to trigger apoptosis or induce a higher level of apoptosis is not known. Notably, a small part of the HN gene (443 bp) was able to induce apoptosis in CEF cells but at low level (26). In the present study, the complete HN gene that was used to induce apoptosis is 1996 nucleotides long and encodes a predicted HN protein of 581 amino-acids as previously reported (57). In adenovirus type 5 E1A protein, only one of the different domains was responsible for apoptosis induction (58). The trans-membrane portion of the Sindbis virus surface glycoprotein E1 and E2 can induce apoptosis (59). HIV envelope protein with truncated cytoplasmic domain was sufficient to induce apoptosis (60). HN protein-induced apoptosis may be dependent on host cellular membrane localization (53). In addition, a membrane anchored HN protein was shown to have a better antitumor effect than the cytoplasmic or secreted protein (61).

Induction of apoptosis is possibly independent of the virus pathotype. Apoptosis was induced by very virulent NDV AF2240 strain as shown in the present study in agreement with other reports (27,28). Apoptosis was also induced by avirulent, non-lytic NDV Ulster strain (53) and by moderately pathogenic Beaudette C NDV strain and avirulent NDV LaSota strain (16). In the present study, we found that MCF-7 cells were more sensitive to NDV AF2240 strain. MCF-7 cells were also reported to be sensitive to NDV Ulster strain (72% apoptotic cells at 72 h pi) (53). However, MCF-7 cells were shown to be resistant to the recombinant NDV LaSota and Beaudette C strains (16). The aforementioned suggestion assumed that HN protein binding to the host cell surface receptor may be responsible and sufficient to trigger apoptosis in NDV-infected cells. However, this suggestion is not certain and how the binding transduces stimuli into the cell or how it activates apoptotic pathways remains unclear and requires further investigations. Similarly, the binding of the reovirus surface attachment protein sigma-1 to sialic acid receptor potentiated virus-induced apoptosis (51) and the binding of HIV surface envelope glycoprotein to its receptor triggered apoptosis (60).

NDV AF2240 HN protein-induced apoptosis may also be the result of the modification of the highly ordered lipid rafts following the accumulation of HN glycoprotein at the MCF-7 cell membranes. These changes have previously been reported to be associated with apoptosis such as in the case of the accumulation of gp120 protein on the membrane of HIV-infected cells (62) and the rabies virus G protein on cell membranes (56).

Another explanation for HN-induced apoptosis may include the involvement of oxidative stress where it was previously reported that an increase in oxidative stress in CEF cells transfected with recombinant HN was observed (26). An earlier study suggested that oxidative stress induced by NDV may be involved in apoptosis induction (63).

The mechanism of HN glycoprotein-induced apoptosis, the binding of HN glycoprotein to cell receptor and the accumulation of HN glycoprotein in cell membrane-induced apoptosis are not known. HN glycoprotein of NDV AF2240 strain may be responsible either alone or in association with other viral proteins for inducing apoptosis and since the oncolytic activity of NDV is mediated by apoptosis (16,17) HN glycoprotein may then also be responsible for the oncolytic activity of NDV as suggested elsewhere (22–24,52). Since HN glycoprotein mediates NDV cytopathogenecity (10) and the difference in NDV cytotoxicity and probably its oncolytic activity is due to differences in the nucleotide sequence of the HN gene and the amino acid sequence of the HN protein (16), we suggest that apoptosis induced by HN is part or an end of the cytopathogenic process. Previous evidence supports this suggestion. First, NDV-induced apoptosis may be involved in NDV cytotoxicity (64). Second, NDV-induced cytopathic effect in infected cells is the result of NDV-induced apoptosis (65). Third, NDV antitumor cytotoxicity may be due to the expression of TRAIL-well known cytotoxic molecule induced by HN expression (20,22). The correlation between pathogenicity and oncolysis properties of NDV was previously reported (66).

As a result, HN may represent a good candidate for antitumor therapy if further improvements are included. In fact, the antitumor property of HN glycoprotein has already been exploited for antitumor therapy (24,67,68). In conclusion, HN protein expression alone induced apoptosis in MCF-7 cells but it was less efficient than the parental NDV AF2240 strain in inducing apoptosis. Therefore, NDV AF2240 strain-induced apoptosis in MCF-7 cells was most probably mediated by HN protein expression alone. Unless the apoptosis-inducing property of HN protein is further improved, NDV AF2240 strain was shown to be more efficient in inducing apoptosis and probably more oncolytic than the recombinant HN protein. Currently, we are focusing on the elucidation of the molecular mechanism of HN inducing apoptosis in MCF-7 cells via investigation of the HN interaction with the cellular membrane proteins and molecular machinery of MCF-7 cells.

## Abbreviations

ATCC: American Type Culture Collection
HAU: hemagglutination unit
HN: hemagglutinin-neuraminidase
IFN-α: interferon alpha
NDV: Newcastle disease virus
NP: nucleoprotein
pi: post infection
RT-PCR: reverse transcription-polymerase chain reaction
TNF: tumor necrosis factor
TRAIL: TNF-related apoptosis-inducing ligand
wt: wild-type
